# Supplementary material for: Recent Trends and In-Hospital Mortality of Transarterial Chemoembolization (TACE) in Germany: A Systematic Analysis of Hospital Discharge Data between 2010 and 2019
Source: Cancers (Basel). 2022 Apr 22;14(9):2088. doi: 10.3390/cancers14092088 (PMC9100764; doi:10.3390/cancers14092088)
Supplement: Supplementary file 1 [file cancers-14-02088-s001.zip › cancers-1666957-supplementary.pdf]

cancers

Article

Recent Trends and In-Hospital Mortality of Transarterial Chemoembolization (TACE) in Germany: A Systematic Analysis of Hospital Discharge Data between 2010 and 2019

Sarah Krieg, Tobias Essing, Andreas Krieg, Christoph Roderburg, Tom Luedde and Sven H. Loosen

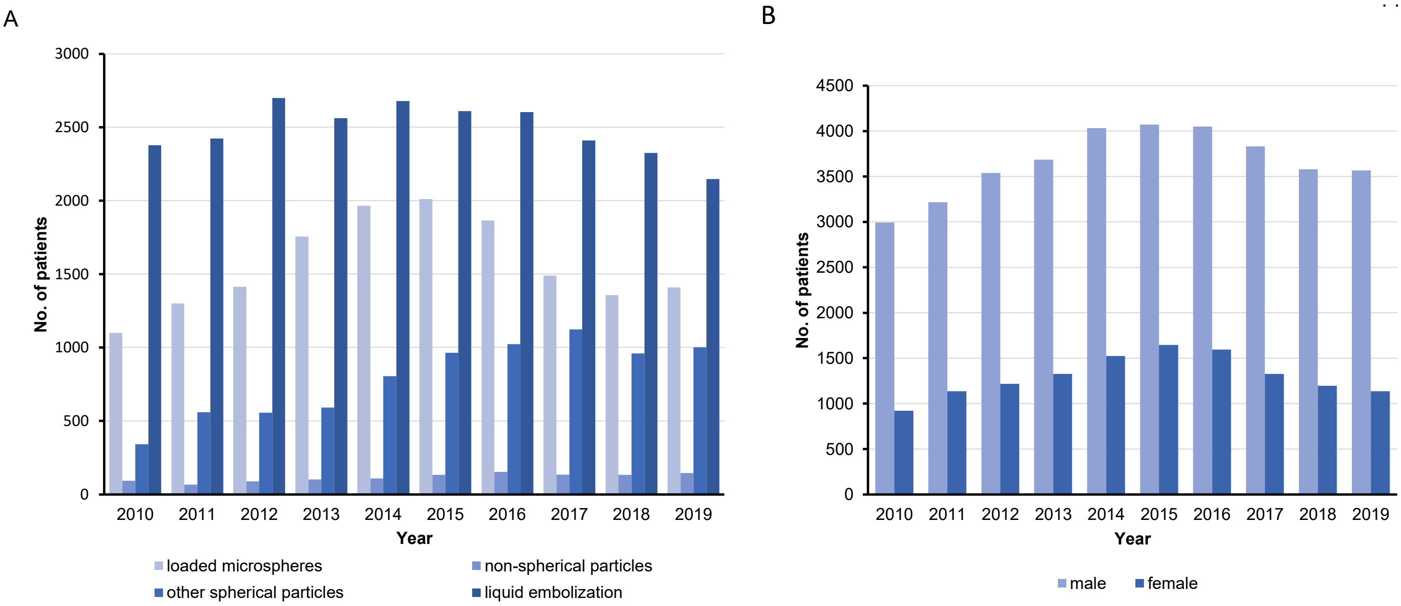

Figure S1. Prevalence and gender distribution for TACE in Germany between 2010 and 2019. (A) Number of TACE procedures performed according to the different embolization agents between 2010 and 2019. (B) Gender distribution of AP patients does not significantly change between 2010 and 2019.

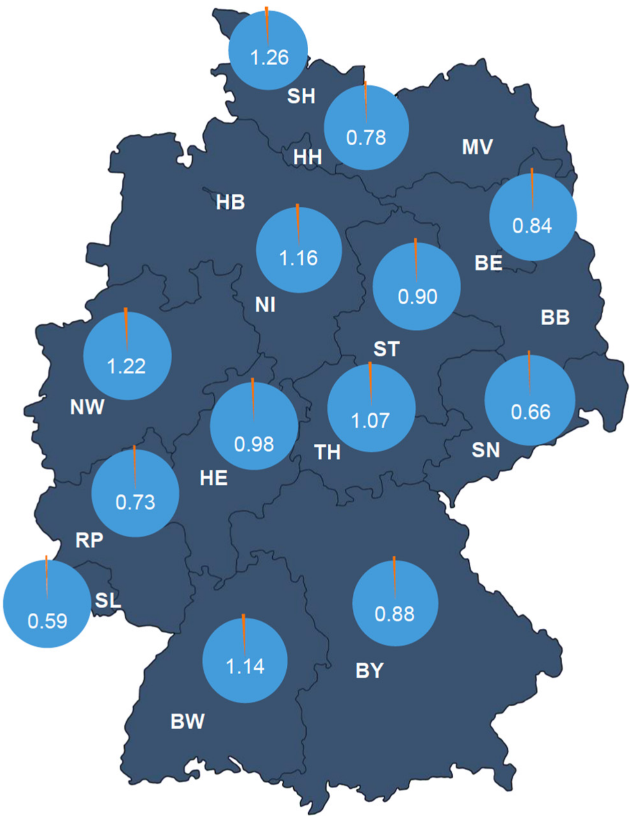

Figure S2. In-hospital mortality following TACE between 2010 and 2019. There is no significant difference in in-hospital mortality between federal states (BB: Brandenburg, BE: Berlin, BW: Baden-Württemberg, BY: Bavaria, HE: Hesse, HB: Bremen, HH: Hamburg, MV: Mecklenburg-Western Pomerania, NI: Lower Saxony, NW: North Rhine-Westphalia, RP: Rhineland-Palatinate, SH: Schleswig-Holstein, SL: Saarland, SN: Saxony, ST: Saxony-Anhalt, TH: Thuringia). For three states, no data were available for analysis (HB, BB and MV).
